# Clinical trial of manual therapy in the treatment of chondromalacia patellae

**DOI:** 10.1097/MD.0000000000033945

**Published:** 2023-06-16

**Authors:** Yan Cai, Ying Deng, Liang Ou, Yuxing Guo, Yanxing Guo

**Affiliations:** a Hunan University of Chinese Medicine, Changsha, China; b The Affiliated Hospital of Hunan Academy of Traditional Chinese Medicine, Changsha, China.

**Keywords:** chondromalacia patellae, clinical trial, manual therapy

## Abstract

Chondromalacia patellae (CP) is a common and main cause of knee pain, the prevalence of the disease in the general population is as high as 36.2%, especially in middle-aged patients aged between 30 and 40 years (up to 50%). The use of manual therapy (MT) to dredge the meridians and muscles around the knee joint and stimulate the relevant acupoints can play vital roles in relieving pain and improving function. The purpose of this study is to assess the effectiveness, safety and further comprehensively, completely and multi-dimensionally explain the mechanism and treatment advantages of MT for CP.

**Methods:**

A prospective randomized controlled clinical trial design was used to study the efficacy and safety of MT in the treatment of CP. One hundred and twenty cases of CP patients will be recruited and randomly divided into experimental group and control group according to 1:1. The control group: sodium hyaluronate; experimental group: MT added on the basis of the control group. Both groups will receive standard treatment for 4 weeks and followed up for 3 months. And at the same time, pay attention to its efficacy and safety indicators. Observation indicators include: the visual analogue scale pain score; the Western Ontario and McMaster Universities Arthritis Index scores; the Lysholm scores, and Bristol scores, adverse reactions, etc. Data analysis was performed using SPSS 25.0 software.

**Discussion:**

This study will precisely evaluate the effectiveness and safety of MT in the treatment of CP. The results of this experiment will provide more reliable clinical basis for the selection of MT for patients with CP.

## 1. Introduction

Chondromalacia patellae (CP), also known as patellofemoral pain syndrome, anterior knee pain syndrome, patellofemoral joint syndrome, etc., was first proposed by Aleman.^[1]^ It is a degenerative disease of patellar cartilage, which is a common and main cause of knee pain. Most patients with this disease have different degrees of poor patellofemoral joint force line and (or) abnormal patellar motion track, making the lateral patella perennial high pressure, which can lead to the secretion of pain-causing inflammatory factors such as interleukin-6, causing pain and subsequent patellofemoral articular cartilage softening until gradual denudation. The prevalence of the disease in the general population is as high as 36.2%, especially in middle-aged patients aged between 30 and 40 years (up to 50%). The prevalence rate in women is higher, about twice that in men.^[2]^ This disease often occurs after strenuous exercise, which can lead to soft legs, joint swelling and pain, restricted movement and other symptoms. The pain is obvious after going upstairs and downstairs, climbing, squatting, kneeling and even sitting for a long time, which seriously affects the quality of life.^[3,4]^ Hence, CP has become a major global public health problem, and the effective treatment is of great significance.

CP is generally treated conservatively in the early stage of the disease, while surgical treatment is suitable for advanced patients. Through methods including cartilage transplantation and total knee replacement, the normal tissue structure around the patella can be restored and reconstructed, and finally the purpose of treating the disease can be achieved.^[5,6]^ The consensus released by the International Patellofemoral Research Center in 2018 believes that the key treatment of CP is exercise therapy and physical intervention,^[7]^ and its management should include an individual, multi-modes exercise and treatment method.^[8]^ Among all the treatment methods, manual therapy (MT) has occupied a certain advantage in clinical application due to its simple operation, obvious effect, small side effects, high safety and good economic effect.

Based on modern western medical theory, MT techniques include joint mobilization, manual stretching and trigger point dry needle. Many studies have found that joint mobilization is effective in pain reduction, while the most effective joint mobilization is aim at the knee joint complex directly rather than at the lumbar-pelvic region or other surrounding joints, although lumbar-pelvic manipulation can also improve patients’ pain and function.^[9–11]^ The use of traditional Chinese medicine massage to dredge the meridians and muscles around the knee joint and stimulate the relevant acupoints can also play vital roles in relieving pain and improving function.

In recent years, studies on the effect of MT for CP have increased at home and abroad, but its clinical efficacy is still unclear. Therefore, the purpose of our study is to precisely evaluate the advantages and disadvantages of MT in the treatment of CP in this randomized controlled trial.

## 2. Materials and methods

### 2.1. Study design

**Figure 1.**
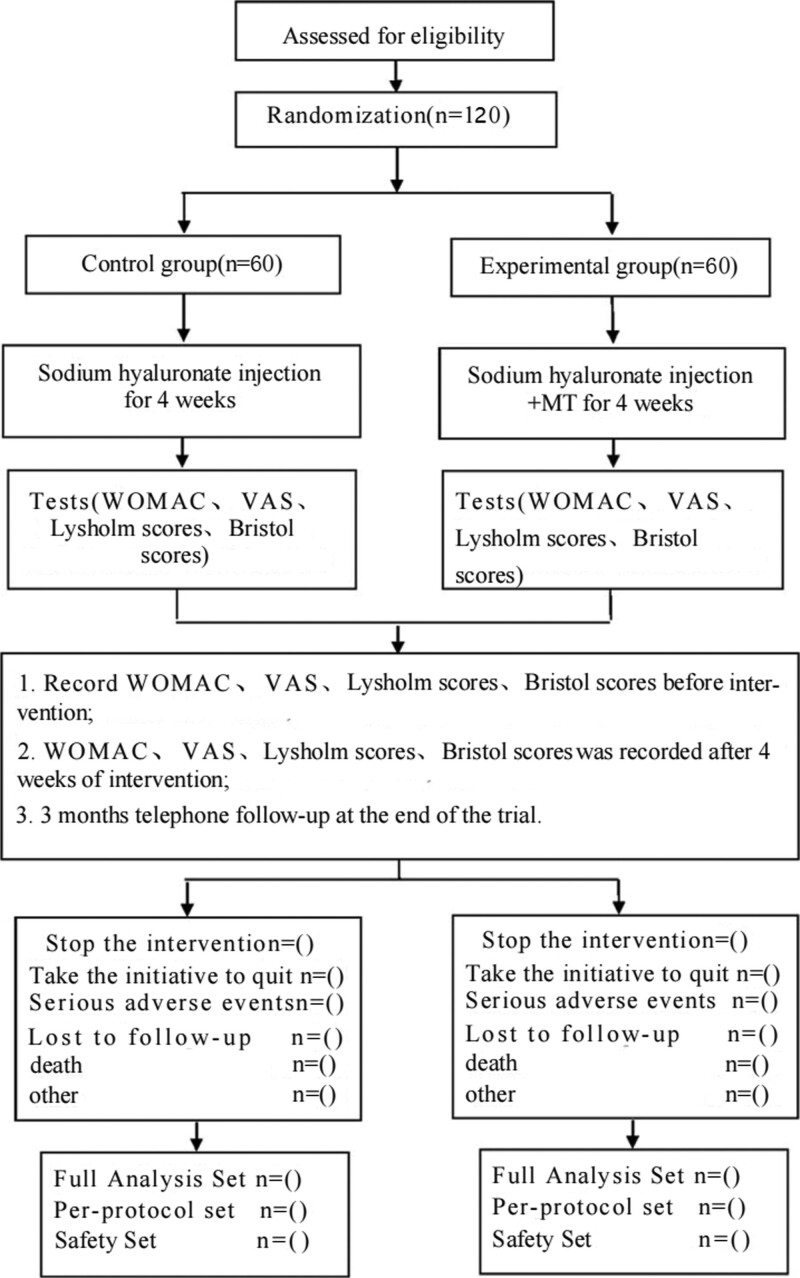
Study flow chart.

This is a prospective randomized controlled clinical trial studying the effects of MT on CP. This protocol follows the latest Consolidated Standards of Reporting Trials (2017) (flowchart is shown in Fig. 1) and Standard Protocol Items: Recommendations for Interventional Trials 2013 statement.

### 2.2. Ethics and registration

This research scheme is approved by the Clinical Research Ethics Committee of our hospital. This experiment has been registered om Chinese Clinical Trial Registry (registration number: ChiCTR2300068655). Before randomization, all patients needed to sign an informed consent form, and only patients who sign informed consent will be enrolled in the study. During the test, they are free to choose whether to continue the test at any time.

### 2.3. Patients

#### 1.2.3. Inclusion criteria.

Those who meet the diagnostic criteria of CP; age: ≥18 and ≤65 years old, regardless of gender; within 3 months before enrollment, they did not receive joint cavity injection treatment and did not take any drugs to treat CP; those who have certain communication and understanding ability, and can express their own will correctly; no other soft tissue and bone diseases of the knee joint; those who voluntarily accept corresponding treatment and can cooperate; no other serious nervous or mental diseases; and those who have signed the informed consent.

#### 2.2.3. Exclusion criteria.

Patients who suffer from acute systemic illness or acute swelling; patients who have severe impairment of limb function; patients with severe heart, lung, liver and kidney dysfunction; patients with local skin infection or ulceration; patients combined with severe mental illness; patients who are allergic to the drugs used in this study; and patients who are unable to understand the study protocol or unwilling after explanation.

#### 3.2.3. Elimination criteria.

Patients with serious adverse events or serious complications, which is not suitable for the next step; patients with poor compliance, affecting the judgment of efficacy and safety; the disease progresses during the treatment process, and the treatment plan needs to be changed; and for any reason, the subject asked to withdraw from the trial.

### 2.4. Sample size calculation

According to the preliminary clinical trial, the total effective rate of MT combined with sodium hyaluronate was 93.4%, and that of sodium hyaluronate was 66.6%. Use PASS15.0 to estimate the sample size, adopt a superiority design, in which α = 0.05, β = 0.2, test power = 0.8, number of cases in experimental group: number of cases in control group = 1:1, boundary value = –0.4. According to the software calculation, the total sample size of the 2 groups was 110 cases. Taking into account the clinical shedding rate of about 10%, a total of 120 cases will be finally included. The included patients will be numbered according to the order of treatment and divided into the treatment group and the control group by completely random method, with 60 cases in each group.

### 2.5. Interventions

This study will select patients who meet the study criteria by recruiting them at the hospital. A randomized controlled study will be conducted, in which sodium hyaluronate will be used in the control group and the experimental group will include MT exercise on the basis of the control group. If necessary, the attending doctor may adjust the treatment according to the patient’s condition, and all interventions will be recorded in detail for final outcome analysis. Efficacy assessors are not aware of the study plan, and data statisticians are not involved in study design and implementation. The health of each patient will be assessed before and after the treatment, including observational indicators, and all patients will be followed up by telephone.

#### 2.5.1. Control group.

Patients will be placed in the sitting or supine position, and the knee joint will be kept bent 70° to 90°, sterilized strictly, with sterile towel laid and aseptic operation performed. The lower lateral or inner side of the patella will be used as the conventional puncture point, and the knee joint cavity will be punctured through a 5 mL syringe. If there is joint effusion, it will be drained first, and then 25 mg sodium hyaluronate will be injected (KUNMING BAKER NORTON PHARMACEUTICAI, SALES CO.LTD. [Kunming City, Yunnan Province, China], specification 2.5 mL: 25 mg, CFDA approval number: H20140533); if not, 25 mg sodium hyaluronate can be directly injected into the joint cavity. After injection, help the patient to move the knee joint so that sodium hyaluronate can be evenly coated on the internal surface of the joint. One time/week, 4 times as a course of treatment. Health education should be carried out for patients to avoid acute jumping, walking up and down stairs, climbing mountains and other activities that damage joints, and remind them to pay attention to the warmth of joints as well. Pay attention to communicate with patients, be aware of their psychological and emotional changes, avoid or relieve anxiety, depression, and other adverse emotions of the elderly patients.

#### 2.5.2. Treatment group.

The treatment group will be given MT on the basis of conventional treatment. Manual therapy is jointly completed by 2 experienced doctors, it lasts for about 20 minutes per time, once a day, and lasts for 4 weeks in total.

### 2.6. Outcomes

#### 2.6.1. Observation indicator.

Improvement in joint pain and function is the main observation item, and which is respectively assessed by the visual analogue scale pain score or a comprehensive evaluation commonly using the Western Ontario and McMaster Universities Arthritis Index scores, Lysholm scores, or Bristol scores.

#### 2.6.2. Efficacy indicator.

Main efficacy indicators: total effective rate: (clinical control + significantly effective number + effective number)/total number of people × 100%. Clinical control: pain and other symptoms disappear, joint activity is normal, integral reduction ≥ 95%; significantly effective: pain and other symptoms disappear, joint activity is not limited, 70% ≤ integral reduction < 95%; effective: pain and other symptoms basically disappear, joint activity is slightly limited, 30% ≤ integral reduction < 70%; and ineffective: no significant improvement in symptoms such as pain and joint activity, integral reduction < 30%.

#### 2.6.3. Adverse events rate.

Including the number of patients who experience any uncomfortable symptoms during treatment (such as dizziness, nausea, etc).

### 2.7. Data collection and management

Data will be collected according to the evaluation criteria before and 4 weeks after the treatment, and each patient was followed up by telephone for 3 months after the end of treatment. It was impossible to collect follow-up information and record the reasons for loss to follow-up. Access to the database is limited to the researchers in this research group.

### 2.8. Study quality control

Throughout the trial, each participant will be monitored for safety. All adverse event occurrences will be referred to the office of the Clinical Research Ethics Committee, which will review the events and determine causal relationship.

### 2.9. Statistical analysis

In this study, SPSS25.0 statistical analysis software (IBM, Amenk, NY) will be used for data analysis. If the measurement data meet the normal distribution, independent-sample *t* test will be used between groups and paired-sample *t* test will be used within groups; if not, non-parametric test will be used and the results will be expressed in quartiles; the count data were tested by chi-square. The incidence of adverse events is compared by chi-square test. *P* < .05 is statistically significant.

## 3. Discussion

CP is one of the common diseases of knee joint. Pain and dysfunction caused by CP severely decreases the quality of life of patients. Currently, the diagnostic standard of CP has not yet formed a unified consensus. There is no effective cure for the treatment of CP, and the effects of surgical treatment which are widely used in clinic, such as cartilage transplantation and total knee replacement, is not superior o non-surgical treatment.^[12,13]^ Moreover, doctors and patients concern constantly about the potential adverse effects of these operation.

At present, MT is one widely used conservative treatment for patients with CP. Manipulative therapy can promote blood circulation and accelerate metabolism, mainly through the following mechanisms to achieve the purpose of treatment: increase the local nutrient supply, and strengthen the “pumping effect” of cartilage tissue itself, which is conducive to the self-repair of the articular cartilage. Eliminate local intra osseous venous stasis, reduce intra ossseous hypertension, and promote absorption of local inflammatory tissue. Exogenous cold pathogen is one of the causes of CP, manipulative treatment can generate heat and disperse cold, and promote local Qi and blood circulation. Promote the mechanical balance of the knee joint, release the adhesion, and improve the stability and functional state of the knee joint.

As far as we know, there has been no standard large-sample clinical study on MT in the treatment of CP, although increasing studies have found that MT has a statistically significant effect on alleviating symptoms of CP with a well-established safety profile. We intend to evaluate its efficacy and safety in a prospective randomized controlled study.

## Author contributions

**Conceptualization:** Yan Cai, Ying Deng.

**Data curation:** Yan Cai, Yanxing Guo.

**Formal analysis:** Yan Cai, Yuxing Guo.

**Software:** Yan Cai, Yuxing Guo.

**Writing – original draft:** Liang Ou, Yanxing Guo.

**Writing – review & editing:** Yan Cai, Ying Deng.
